# Plasmacytoid dendritic cell and myeloid dendritic cell function in ageing: A comparison between elderly and young adult women

**DOI:** 10.1371/journal.pone.0225825

**Published:** 2019-12-12

**Authors:** Marloes van Splunter, Olaf Perdijk, Henriëtte Fick-Brinkhof, Esther G. Floris-Vollenbroek, Ben Meijer, Sylvia Brugman, Huub F. J. Savelkoul, Els van Hoffen, R. J. Joost van Neerven

**Affiliations:** 1 Cell Biology and Immunology, Wageningen University, Wageningen, The Netherlands; 2 Human Nutrition, Wageningen University, Wageningen, The Netherlands; 3 NIZO Food research, Ede, The Netherlands; 4 FrieslandCampina, Amersfoort, The Netherlands; Emory University School of Medicine, UNITED STATES

## Abstract

Ageing is associated with a changing immune system, leading to inflammageing (increased levels of inflammation markers in serum) and immunosenescence (reduced immune cells and reduced responses towards pathogens). This results in reduced vaccination responses and increased infections in elderly. Much is known about the adaptive immune system upon ageing, but less is known about the innate immune system. Therefore, the aim of this study was to compare innate immune function of Toll like receptor (TLR)-mediated responses between elderly and young adult women.

To this end, elderly and young adult women were compared to study the effect of ageing on the relative prevalence and reactivity to TLR-mediated responses of myeloid- and plasmacytoid dendritic cells (mDC, pDC). In addition, TLR expression and inflammatory markers in serum were investigated. Elderly women had reduced numbers of circulating pDCs. In addition, pDCs and mDCs of elderly women responded differently towards TLR stimulation, especially TLR7/8 mediated stimulation was reduced, compared to young adults. In serum, markers involved in inflammation were generally increased in elderly. In conclusion, this study confirms and extends the knowledge about immunosenescence and inflammageing on innate immunity in elderly women.

## Introduction

The ageing population is growing rapidly, and more than 30% of all people are expected to be >65 year old in 2050 compared to 10–20% in 2015 [1]. This is especially the case in Europe, North America and East Asia [1]. Ageing is associated with changes in the immune system. The lifelong history of infections, changes in microbiota composition, diet, physical activity and stress all contribute to decreased immune function in elderly people [2]. Immune deficiency during ageing occurs at two levels: irreversible primary immune deficiency and reversible secondary immune deficiency of which low nutritional status is an example [3]. Immunosenescence can be seen as an example of primary immune deficiency, in which both adaptive immune responses by B and T cells are reduced, as well as responses of the innate immune system.

Much is known about the effect of ageing on the adaptive immune system, as reviewed by Ventura et al [4]. Numbers of naïve T and B cells are declining during ageing, as well as effector memory T cells. Besides, CD8^+^ effector T cells are increased, but change phenotypically (e.g. loss of CD8) and regulatory T cells numbers are increased [4]. In contrast, fewer mature B cells are found upon ageing due to declining numbers of progenitors. Serum levels of IgM and IgD are reduced, while IgG and IgA levels are increasing upon ageing [4]. In addition to this, first line immune defences such as the skin, becoming fragile with age and antibody production by the mucosal immune system, are decreased in elderly [5].

Less is known about the effect of ageing on the innate immune system [6]. In ageing reduced responsiveness to pathogens is observed due to reduced expression and activation of pattern recognition receptors (PRRs), such as Toll like receptors [7]. This results in less phagocytosis of pathogens by myeloid cells, resulting in increased levels of C-reactive protein, IL-6 and TNF-α[8]. One of the best documented examples of immunosenescence is the reduced response to influenza vaccination in elderly, which results in only 33% of the cases in protection of elderly, compared to 59% in adults (16–65 years old) [9]. This is partly caused by the fact that the vaccines are optimized for young adults [10]. Elderly (and children) are most vulnerable to influenza infections[11,12]. In addition, influenza infection is associated with an increased rate of pneumonia and other respiratory illnesses, resulting in higher mortality rates in elderly during influenza epidemics [13,14].

Myeloid dendritic cells (mDCS) and plasmacytoid dendritic cells (pDCs) are two types of human blood DCs that derive from different progenitors and have different functions[15]. MDCs regulate pro-inflammatory responses via inducing T-helper 1 and cytotoxic T lymphocyte responses upon bacterial and viral infections [16]. Plasmacytoid dendritic cells (pDCs) produce type I interferons (IFNs), for example upon Toll like receptor (TLR) 7 mediated activation by influenza virus[10]. Reduced pDCs numbers in the elderly may in part explain the increased occurrence of severe influenza infections in this age group [17].

Inflammageing is another immunological phenomenon associated with ageing. Inflammageing is defined as the increase in inflammatory factors in serum that is seen in ageing [18]. Inflammageing is observed both in diseased elderly as well as healthy centenarians, with high levels of IL-6 being correlated with increased morbidity and mortality [18]. It is hypothesized that after a lifetime of inflammatory immune responses, the immune system in the elderly fails to downregulate these responses, resulting in a low-grade chronic inflammation[19]. Whether the increased levels of pro-inflammatory cytokines lead to age-related diseases or not is a delicate tipping point and probably differs per individual [18]. A possible explanation could be the observation seen in mice that during ageing the Treg number increases, resulting in a suppression of T cell responses (e.g. IFN-γ production) and can subsequently lead to enhanced chronic infections[20]. Inflammageing may relate to the fact that in a relatively short timeframe, during the last century, life expectancy increased far beyond our reproductive age. It has been suggested that the immune system may not have adapted to this elongation of inflammatory exposures[19].

After menopause, pro-inflammatory cytokines (IL-1, IL-6 and TNF-α) are increased in serum and CD4^+^ T helper cells and B cells are decreased in women, resulting in lower humoral immunity. A consequence of the reduced T helper and B cells numbers is higher susceptibility to infections, which could be linked to reduced estrogen levels in postmenopausal women [21]. Estrogen receptors are found on all kinds of innate and adaptive immune cells, including macrophages and dentritic cells[22]. Furthermore, pDCs of postmenopausal women produce less IFN-α compared to pre-menopausal after TLR7/8 stimulation [23,24]. This effect can be inverted by estrogen supplementation, as pDCs of postmenopausal women were shown to produce more IFN-α and TNF-α upon TLR7/8 stimulation after 1 month of estrogen supplementation [24].

The aim of the current study was to compare innate immune function towards TLR-mediated responses between elderly and young adult women. To this end, elderly women and young female adults were compared to study the effect of ageing on the relative prevalence and reactivity of myeloid and plasmacytoid dendritic cells (mDC, pDC) to TLR-mediated responses. In addition, TLR expression and pro-inflammatory markers in serum were compared.

## Material and methods

In this study, we investigated the effect of ageing on the innate immune system of young and elderly women. Elderly women (65–85 years) n = 30 who were participants in the ‘NOBLE intervention study‘. The protocol for NOBLE was approved by the Medical Ethics Committee of Wageningen University, the Netherlands (protocol no. NL57345.081.16), and registered at clinicaltrials.gov (identifier NCT03026244). An additional 15 young women (18–30 years) were recruited as a comparator to the more elderly women from NOBLE. After providing written informed consent, all subjects were screened based on the NOBLE study inclusion and exclusion criteria enlisted hereafter [25]. Subjects were included when they were generally healthy, having a BMI 20–30, not smoking and good mental status. Subjects with chronic inflammatory, autoimmune or gastrointestinal diseases or being immune-compromised were excluded from participation. Subjects using hormone replacement therapy, anti-inflammatory drugs (>1x week) or immunosuppressive drugs were also excluded. An overview of the demographic information of the two study groups is given in Table 1.

**Table 1:** Demographics of elderly and young adult study population.

Group	Age in years median (range)	Anti-inflammatory medicine (Y/N)	Vitamine D supplementation before study (Y/N)	Blood pressure or cholesterol medication (Y/N)
Elderly women n = 30	74.5 (69–85)	1/29	13/17	12/18
Young women N = 15	24 (20–29)	1/14	2/13	0/15

### Blood sampling

Blood was collected at the study day for serum storage (10 mL tubes; cat.no. 367895, BD) or for PBMC isolation (K2‐EDTA; 4 x 10mL; cat.no. 367525, BD). Serum tubes were left at room temperature for at least 30 min before centrifugation at 2000 x g 10 min at room temperature. Serum was aliquoted and stored at ‐80°C. PBMCs were isolated within 6 hours using 50 ml Leucosep tubes (227290, Greiner Bio-One) filled with Ficoll plaque plus (17-1440-02, GE Healthcare Life Sciences) according to manufacturer’s protocol. Remaining PBMCs were cryopreserved and stored in liquid nitrogen, protocol: dx.doi.org/10.17504/protocols.io.87phzmn.

### TLR expression in pDCs and mDCs

Isolated PBMCs were stained with a TLR antibody panel (Table 2) to measure the expression of TLR 2, 4, 7 and 9 *ex vivo*. To measure expression of TLRs, 0.4 x10^6^ (young) or 2 x10^6^ (elderly) freshly isolated PBMCs/donor were put in a 96 well plate (NUNC PP Sigma-Aldrich 7116) and per well 200 ul FACS buffer (PBS (Lonza BE17-516Q/12); 2mM EDTA (Merck CBI 108418); 0,5% BSA (Roche 10735086001); 0,01% NaN3 (Merck CBI 822335) was added to wash the cells by centrifuging the plate at 400xg for 3 minutes at 4°C. First, extracellular surface markers (Table 2) including 5 μl Fc block (564220, BD Pharmingen) were stained for 30 minutes on ice covered in aluminium foil and washed twice with cold PBS. Cells were stained with Fixable Viability Dye FVD520 (65086718, Ebioscience) in PBS and incubated for 20 minutes in the fridge, followed by washing the cells FACS buffer. Afterwards cells were permeabilized by adding IC fixation buffer (00-8222-49, Ebioscience) to each well and incubated for 30 minutes at room temperature, followed by washing twice in Perm buffer (00-8333-56, Ebioscience). The intracellular antibody mix (Table 2) in Perm buffer was incubated for 20 minutes in the fridge, followed by washing the cells twice in Perm buffer. Cells were resuspended in 300 ul FACS buffer and measured for 240s on the FACS CANTO II at medium flow rate, threshold 45.000. For the detailed protocol see dx.doi.org/10.17504/protocols.io.87qhzmw. One sample per subject was analysed. As controls the combination of ‘fluorescent minus one’ (FMO) control was used for the TLR expression and the FMO TLR was replaced with their isotype controls. In order to be able to gate pDCs and mDCs, the ‘backbone’ antibodies containing live/death, lineage-2, HLA-DR, CD11c and CD123 were used.

**Table 2:** Antibodies used for TLR expression and intracellular cytokine measurements.

*Antibody*	Fluorchrome	host	isotype	Light chain	company	Catalog number	Panel	Extra/ Intra-cellular
*lineage 2*	FITC	mouse	IgG1	κ	BD	643397	TLR & cytokine	extra
*HLA-DR*	APC-Cy7	mouse	IgG2b	κ	Ebioscience	47-9956-42	TLR & cytokine	Extra
*CD123*	PE-Cy5	mouse	IgG1	κ	Ebioscience	15-1239-42	TLR & cytokine	Extra
*CD11c*	PE-Cy7	mouse	IgG1	κ	Ebioscience	25-0116-42	TLR & cytokine	Extra
*TLR2*	biotin	mouse	IgG2a	κ	Ebioscience	13992282	TLR	Extra
*TLR2 ic*	biotin	mouse	IgG2a	κ	Ebioscience	13472785	TLR	Extra
*streptavidin*	BV510				BD	563261	TLR	Extra
*TLR 4*	BV421	mouse	IgG1	κ	BD	564401	TLR	Extra
*TLR4 ic*	BV421	mouse	IgG1	κ	BD	562438	TLR	extra
*FVD 520*	efluor520				Ebioscience	65-0867-18	TLR & cytokine	Extra
*TLR7*	PE	mouse	IgG2a		R&D Systems	IC5875P	TLR	Intra
*TLR 7 ic*	PE	mouse	IgG2a		R&D Systems	IC003P	TLR	Intra
*TLR9*	APC	rat	IgG2a	κ	Ebioscience	17909982	TLR	Intra
*TLR9 ic*	APC	rat	IgG2a	κ	Ebioscience	17-4321-81	TLR	Intra
*CD16*	BV510	mouse	IgG1	κ	BD	740203	Cytokine	Extra
*IL-6*	pe	rat	IgG1	κ	Ebioscience	12706982	Cytokine	Intra
*IL-6 ic*	PE	rat	IgG1	κ	Ebioscience	12430183	cytokine	Intra
*IFN-alpha*	V450	mouse	IgG1	κ	BD	561382	Cytokine	intra
*IFN-alpha ic*	V450	mouse	IgG1	κ	BD Horizon	561504	cytokine	intra
*TNF-alpha*	apc	mouse	IgG1	κ	Ebioscience	17734982	Cytokine	Intra
*TNF-alpha ic*	APC	mouse	IgG1	κ	Ebioscience	17-4714-41	Cytokine	Intra

Antibody mixes were made for extra-cellular or intra-cellular staining. Ic = isotype control

Flow cytometry data analysis was performed by using FlowJo software (version 10 TreeStar, Inc.) and gating for mDCs and pDCs was performed as is shown in S1 Fig, in line with Panda et al[16]. Data were exported as median fluorescent intensity for either all pDCs or mDCs per TLR.

### Intracellular cytokine measurement in pDCs and mDCs

**Fig 1:**
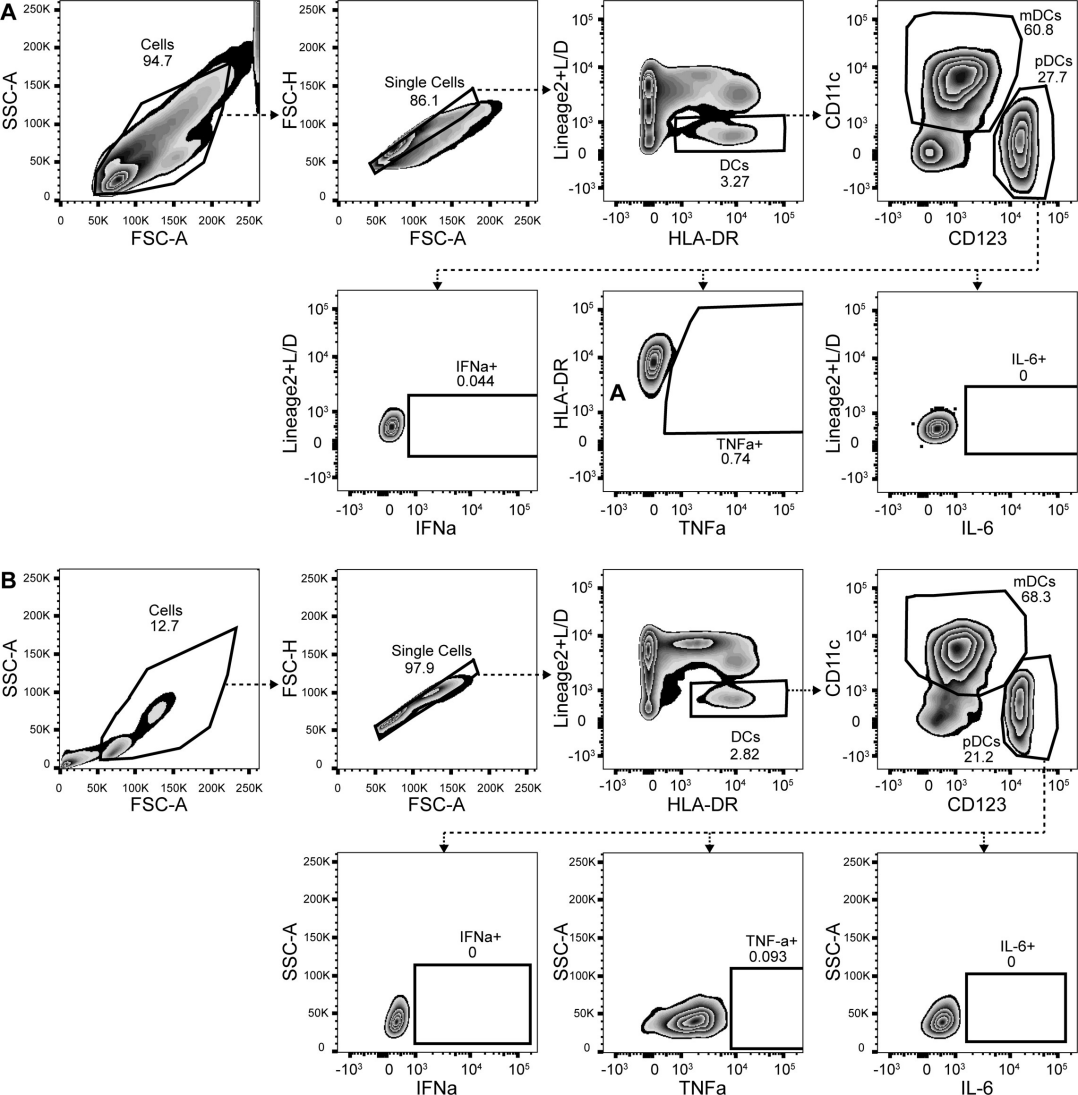
**Gating strategy of intracellular cytokine production** by a donor from the elderly population (171/002) (A) or a donor from the young adult population (171/young/003) (B) in steady state (RPMI).

In order to measure intracellular cytokines 0.4 x10^6^ (young) or 2 x10^6^ (elderly) PBMCs were stimulated in a 12-well plate (CLS3513-50ea, Sigma-Aldrich) (total volume 1 ml) for three hours with RPMI-1640 (Be112-115F, Lonza), TLR1/2 ligand PAM3CSK4 (PAM) 10 μg/ml (L2000, EMC microcollections), TLR4 ligand Ultra-pure LPS 0,1 μg/ml (3pelps, Invivogen), TLR7/8 ligand R848 3 μg/ml (TLRL-R848-5, Invivogen) or TLR9 ligand CpG 3 μg/ml (TLRL-2216-1 (class ‘A’), Invivogen). All TLR stimulations were done in the presence of Brefeldin A (B7651, Sigma-Aldrich) in RPMI-1640 with 5% human AB serum (H4522, Sigma Aldrich). Afterwards, cells were harvested by pipetting and stained in the same way as described for the TLR staining. Cells were resuspended in 250 ul FACS buffer and one sample per stimulation per subject was measured for 200s with FACS Canto II. For the detailed protocol see dx.doi.org/10.17504/protocols.io.87qhzmw. Flow cytometry data analysis was performed by using FlowJo software (version 10 TreeStar, Inc.) and gating was performed as is shown in Fig 1. As controls, the combination of ‘fluorescent minus one’ (FMO) control was used for the intracellular cytokine production and intracellular cytokines were replaced with their isotype controls, see S2 Fig. In order to be able to gate pDCs and mDCs, the ‘backbone’ antibodies containing live/death, lineage-2, HLA-DR, CD11c and CD123 were used. Gating of DCs and pDC and mDCs was adapted per donor, other gates were set based on FMO + ic controls or negative versus positive population in most donors. Data were exported as percentage cytokine-positive pDCs or mDC as percentage of all pDCs or mDCs.

### Cytokine and pro-inflammatory marker measurements in serum

In serum, IL1β (558279, BD Pharmingen), TNF-α (560112, BD Pharmingen), IL-6 (558276, BD Pharmingen) sCD106 (sVCAM-1; 560427, BD Pharmingen), sCD54 (ICAM-1; 560269, BD Pharmingen) and IL-10 (558274, BD Pharmingen) were measured in triplo by cytometric bead array, according to manufacturer’s protocol. Beads were measured for 50 seconds at high speed using a FACS Canto II. Furthermore, IL-1Ra (CHC1183, Thermo Fisher) was measured by ELISA and C-Reactive Protein (CRP) was measured using C-Reactive Protein kit ELISA (Ebioscience, 88-7502-28) according to manufacturer’s protocol.

### Statistical analysis

Statistical analysis was performed by using IBM SPSS Statistics version 23. Data were tested for normal distribution using Shapiro-Wilk test. The cytokine production (% of all pDCs or all mDC) of pDC and mDCs per stimulation (RPMI, PAM, LPS, R848 and CpG) were analysed by MANOVA with a pairwise comparison using bonferroni correction. To obtain normally distributed data, percentages were logit-transformed after which outliers (>2SD) were removed. This resulted in the removal of 0–3 outliers in the elderly study group per stimulation and 0–2 outliers in the young study group per stimulation, see S6 Fig. For TLR 2, 4, 7 and 9 expression the median MFI was analyzed per cell type (pDC or mDC). The median MFI was 10log transformed to obtain normal distributed data. A MANOVA-analysis was performed per cell type (pDC or mDC) with a pairwise comparison using bonferroni correction. Cytokine levels (pg/ml) were 10log transformed or rank transformed (in case of IL1-β, TNF-α and sICAM) to obtain normal distributed data. As the value 0 cannot be 10log transformed, this value was artificially set at 0,001 to obtain a value after transformation. MANOVA-analysis was performed with a pairwise comparison using bonferroni correction.

## Results

### pDC numbers, but not mDC numbers, in peripheral blood relatively decreased upon ageing

**Fig 2:**
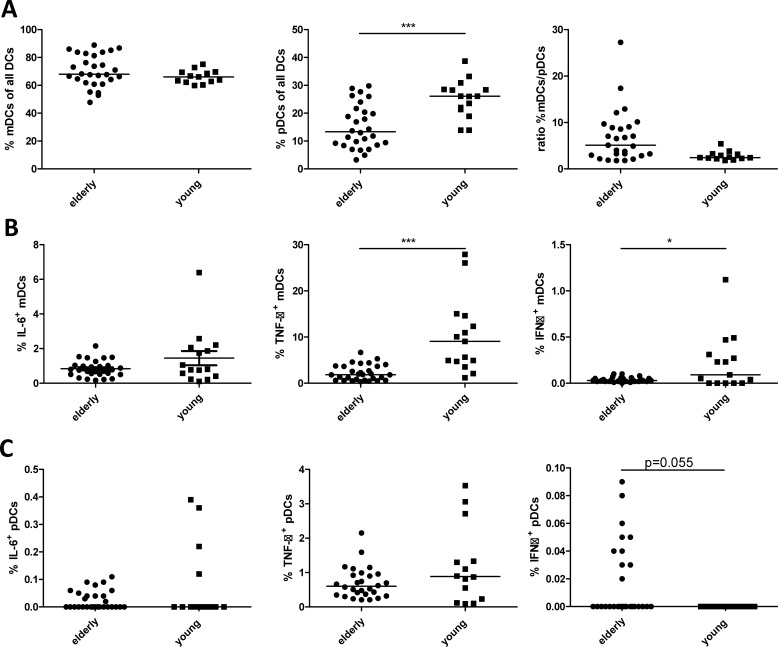
**Percentage of pDCs and mDCs and ratio %mDC/%pDCs in elderly and young adults (A) and percentage of mDCs (B) and pDCs (C) positive for intracellular cytokine production of IL-6, TNF-α and IFN-α in steady state (RPMI).** Data shown as dotplot with median, outliers with >2SD based on transformed data were removed. Elderly n = 30; young n = 15 donors. Statistics were done with logit transformed data using MANOVA with a pairwise comparison and a bonferroni correction. *p<0.05; **p<0.01; *** p<0.001.

To study the relative numbers of mDC and pDC in peripheral blood of elderly and young adult women, PBMC were isolated from freshly drawn blood, and were stained with Lineage markers 2 cocktail, HLA-DR, CD11c, CD123, IFN-α, TNF-α and IL-6. The gating strategy shown in Fig 1 was used to determine the % of pDC and mDC in the total DC population. mDC were defined by expression of Lineage 2^-^ HLA-DR^+^ CD11c^+^ CD123^-^, and pDC were identified by Lineage 2^-^ HLA-DR^+^ CD11c^-^ CD123^+^. In addition to pDC and mDC, a CD11c^-^CD123^-^ double negative cell population was observed. The relative percentage of pDCs and mDCs as part of the total DC population was determined. As shown in Fig 2, the relative percentage of pDCs in elderly women was significantly lower compared to pDC in young adults. In contrast, the relative percentage of mDCs between elderly and young adults did not differ significantly. As a result, the relative percentage of the double negative DC population was higher in the elderly total DC population. To correct for this, the ratio between %mDC and %pDCs was taken. The ratio %mDC/%pDCs, with a mean value of 6.97±6.06, was higher in elderly than in young adults, which had a mean ratio of 2.91 ±1.26, see Fig 2A. As the relative percentage of mDCs was not different between young adults and elderly, this is suggestive for a lower percentage of pDCs of all DCs in elderly, although absolute numbers were not determined with counting beads. Furthermore, the spontaneous production of intracellular cytokines by mDCs (Fig 2B) and pDCs (Fig 2C) was determined in unstimulated PBMC. A higher number of mDCs of young adults produced TNF-α or IFN-α compared to elderly. In contrast, the percentage of pDCs of elderly producing IFN-α was higher compared to young adults, whereas no differences were observed for IL-6 and TNF-α positive pDCs between young adults and elderly.

### Age-related differences in intracellular cytokine production of pDCs in response to TLR stimulation

It was investigated how pDCs respond to TLR stimulations. To this aim, pDCs were stimulated with TLR1/2 ligand Pam3CYSK4 (Pam), TLR 4 ligand LPS, TLR 7/8 ligand R848 and TLR 9 ligand CpG, and intracellular production of TNF-α, IL-6 and IFN-α was determined as described in materials and methods. The gating strategy to detect intracellular cytokine production of pDCs upon TLR stimulation is shown in S1 Fig and of the flow cytometric controls (fluorescence minus one (FMO) and isotype control) in S2 Fig. The relative percentages of pDCs did not change after TLR stimulation, which indicates that the markers used for pDC gating do not change upon stimulation, see S3A Fig. Therefore, the relative percentages of pDCs upon TLR stimulation were still lower in elderly compared to young adults.

When pDC were stimulated with TLR ligands, the number of TNF-α^+^ pDCs was highest for all stimulations. The highest percentage of IFN-a^+^ pDC was detected after stimulation with R848, while IL-6^+^ pDC were detected in comparable numbers upon stimulation with Pam, LPS and R848.

**Fig 3:**
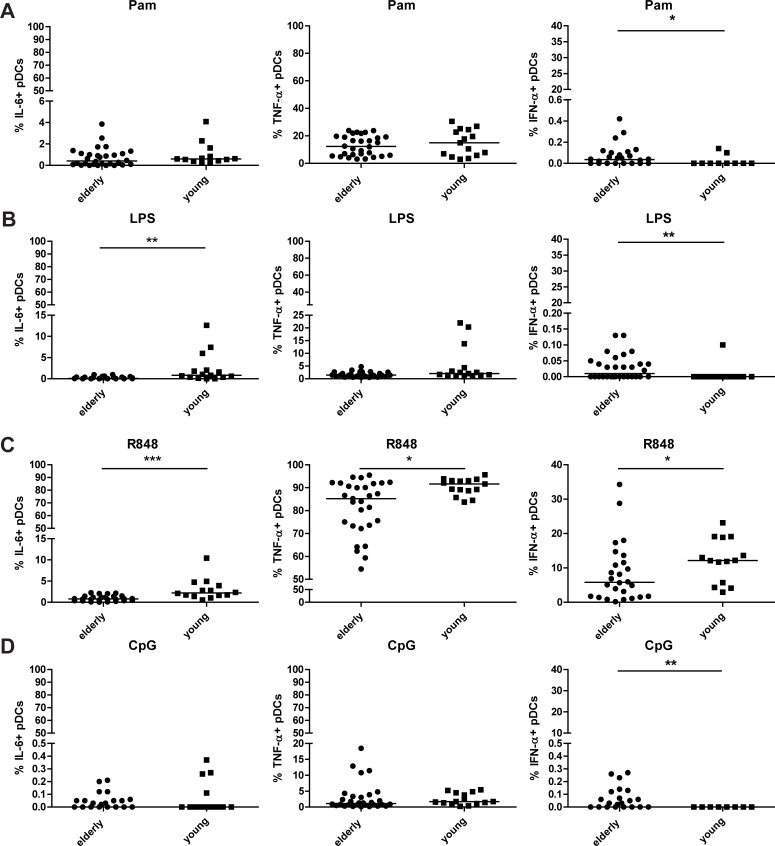
**Intracellular cytokine production (IL-6, TNF-**α **and IFN-**α**) of pDCs upon TLR1/2 (A), TLR4 (B), TLR 7/8 (C) and TLR9 (D) stimulation in young versus older adults.** Data shown as dot plots with median, every dot represents a donor, outliers with >2SD based on transformed data were removed. Elderly n = 30; young n = 15 donors. Statistics were done with logit or rank transformed data using MANOVA per TLR stimulation with a pairwise comparison and a bonferroni correction. *p<0.05; **p<0.01; *** p<0.001.

R848 was the only stimulus that induced strong IFN-α production by pDC. This response was significantly higher in the young adult women compared to the elderly women tested. For the other stimuli, remarkably, the percentage of IFN-α^+^ pDCs was significantly higher in elderly pDC than in young adult pDC (Fig 3A–3D for TLR stimulations other than TLR7/8 stimulation, this may partly be explained by a higher spontaneous production of IFN-α in pDC in elderly women (Fig 2C). The biological relevance of the IFN-α production of pDCs upon stimulation other than R848 is questionable as the percentage of IFN-α^+^ pDCs is below 1%, whereas after R848 stimulation up to 34% are IFN-α^+^ pDCs.

Upon TLR4 and TLR7/8 stimulation, young adults had significantly more IL-6^+^ pDCs compared to elderly (Fig 3B and 3C). Furthermore, the number of TNFa^+^ pDC after TLR7/8 stimulation with R848 was also higher in the young adults.

Taken together, in general, pDCs of young adult women responded more to TLR stimulation than elderly women. This was demonstrated most prominently after stimulation with TLR 7/8, with statistically significant higher percentages of all cytokine-producing pDC in the young adult women. The fact that elderly women also had relatively lower numbers of circulating pDC indicates that this is an underestimation, as the data are represented as % of pDC.

### Age-related differences in intracellular cytokine production of mDCs upon TLR stimulation

The effects of ageing on the percentage of mDCs were less prominent than the effects shown for pDCs. Only after stimulation with TLR4, elderly had a significantly higher percentage of mDCs (S3B Fig) compared to young adults. However, this did not seem to affect differences in intracellular cytokine production, see below.

**Fig 4:**
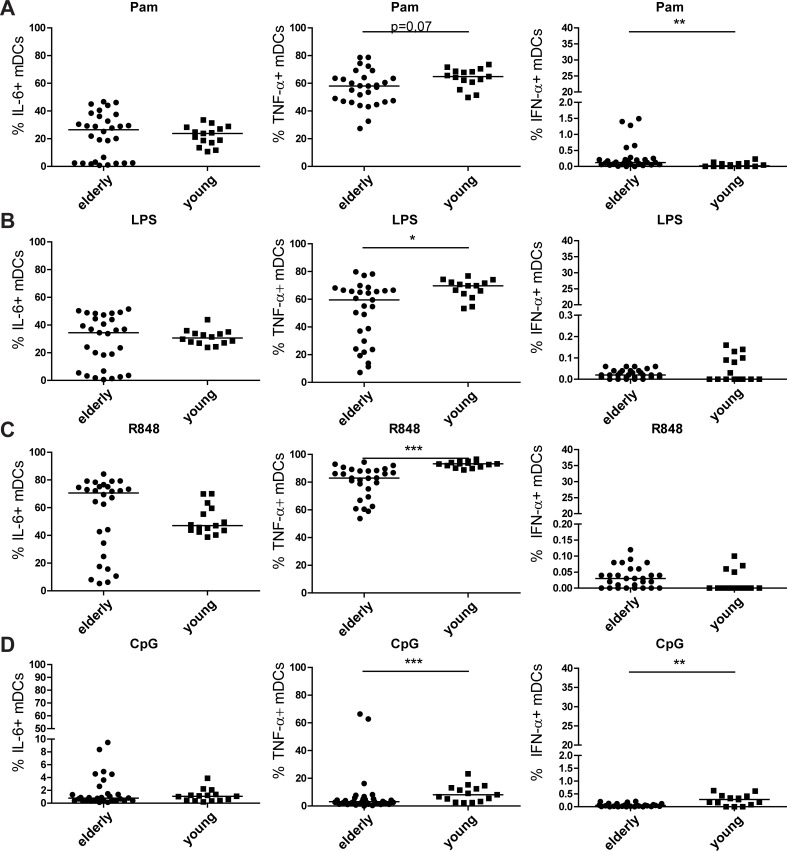
**Intracellular cytokine production (IL-6, TNF-**α **and IFN-**α**) of mDCs upon TLR 1/2 (A), TLR 4 (B), TLR 7/8 (C) and TLR 9 (D) stimulation in young versus older adults.** Data shown as dot plots with median, every dot represents a donor, outliers with >2SD based on transformed data were removed. Elderly n = 30; young n = 15 donors. Statistics were done with logit or rank transformed data using MANOVA per TLR stimulation with a pairwise comparison and a bonferroni correction. *p<0.05; **p<0.01; *** p<0.001.

After stimulation with the different TLR stimuli, only the number of TNF-α^+^ mDCs was significantly higher in young adults after LPS, R848 and CpG stimulation, while after Pam stimulation TNF-α^+^ mDCs tended to be higher (p = 0.07) in young adults compared to elderly, see Fig 4.No significant differences in IL-6^+^ mDCs were observed.

In contrast, elderly had relatively higher percentages of IFN-α^+^ mDCs compared to young adults upon TLR1/2 stimulation (Fig 4A). No differences were observed between elderly and young adults in IFN-α^+^ mDCs after TLR4 and TLR7/8 stimulation. In contrast to pDCs and TLR1/2 stimulation of mDCs, young adults had higher percentages of IFN-α^+^ mDCs when stimulated with TLR9 ligand CpG (Fig 4D). In all cases, the number of IFN-α^+^ mDCs was very low.

In conclusion, young adults had more TNF-α^+^ mDCs compared to elderly upon all TLR stimulations, and more IFN-α^+^ mDCs when stimulated with TLR 9 ligand CpG. However, for the latter this may be partly explained by the increased spontaneous production of TNF-α and IFN-α by mDC as shown in Fig 2B.

### Pro-inflammatory markers sICAM, sVCAM and TNF-α elevated in serum of elderly

To determine the inflammatory status of the elderly and young adult women, a panel of pro-inflammatory markers that are known to be associated with ageing [2] was measured in serum samples. The concentrations of pro-inflammatory markers soluble ICAM, soluble VCAM and TNF-α were significantly higher in sera of elderly compared to sera of young adults, see Table 3. Furthermore, the concentration of IL-10 was also increased in elderly women. IL-1Ra showed a trend (p = 0.06) to be decreased in elderly women. No differences in IL-6, IL-1β and CRP levels were observed.

**Table 3:** Concentration of pro-inflammatory markers in serum in median pg/ml with range (minimum to maximum value) in elderly (n = 30) and young adults (n = 15) women.

	Elderly (pg/ml)	Young adults (pg/ml)	Statistically different
IL-6	3.77 (0.55–10.54)	2.31 (0.00–8.17)	NS
IL-1β	0.22 (0.00–3.59)	0.02 (0.00–1.78)	NS
IL-10	2.93 (0.949–5.71)	1.47 (0.53–5.34)	**
TNF-α	13.57 (0.00–66.93)	0.00 (0.00–48.99)	**
sICAM ng/ml	84.38 (0.00–281.81)	15.10 (0.00–180.48)	***
sVCAM ng/ml	258.48 (104.14–573.42)	153.13 (46.49–328.07)	***
IL-1Ra	58.93 (13.73–3715.71)	87.40 (46.10–179.07)	P = 0.06
CRP (ng/ml)	538 (12–12456)	662 (52–29200)	NS

Statistical analysis was performed using 10 log or rank transformed data by MANOVA with pairwise comparisons using bonferroni correction.

* p<0.05

** p<0.01

*** p<0.001.

ND not determined, statistical analyses not performed as too many values were 0 pg/ml. NS no significant differences.

## Discussion

The results presented here show that elderly women have reduced relative numbers of circulating pDCs. In addition, pDCs and mDCs of elderly women are less responsive to TLR stimulation, especially TLR7/8 mediated stimulation, compared to pDC and mDCs of young adults. In serum, markers involved in inflammation are increased in elderly compared to young adults.

Immune responses are not equal between men and women[16,23]. TLR7 is X-linked which might explain why women have better TLR7-mediated responses [22]. Therefore, we compared young adult women with postmenopausal elderly women.‬‬‬‬‬‬‬‬‬‬‬‬‬‬‬‬‬‬‬‬‬‬‬‬‬‬‬‬‬‬‬‬‬‬‬‬‬‬‬‬‬‬‬‬‬‬‬‬‬‬‬‬‬‬‬‬‬‬‬‬‬‬‬‬‬‬‬‬‬‬‬‬

Ageing is associated with changes in the immune system, leading to inflammageing and immunosenescence[18,19]. One aspect of immunosenescence is that immune cell numbers decline. Elderly women in this study had relatively less pDCs compared to young adult women, which is in line with many other studies[10,16,17,23,26–30], although some studies detected no difference upon ageing [31,32]. Reduced numbers of pDCs could lead to increased TLR7/8 mediated infections, such as influenza and RSV infection [33]. The relative percentage of mDCs in elderly women was similar to young adults in steady state, in agreement with other studies [17,31], although some studies observed a decrease in mDCs percentages upon ageing[27,32]. It is important to look not only to the percentage of pDCs and mDCs but also to the ratio between the two, as there is a third category of double negative CD11c^-^ CD123^-^ cells in the current gating strategy that changes between the two age groups (see Fig 1). Based on FSC/SSC back-gating we hypothesize that these cells could be a mixture of CD16^+^CD56^dim^NK cells, BCDA-3^+^ mDCs or CD34^+^ DC-like cells [30,34,35]. Future research is needed to further investigate the function of this population in ageing.

Besides looking at percentages of pDCs and mDCs upon ageing, their cytokine production in steady state and upon TLR stimulation was also investigated. In steady state, young adult women had more TNF-α^+^ and IFN-α^+^ mDCs, but comparable IL-6^+^ mDCs, while elderly women seemed to have more IFN-α^+^ pDCs, but comparable levels of TNF-α^+^ and IL-6^+^ pDCs in steady state. These data partly confirm the study of Panda et al., who also observed higher levels of TNF-α^+^ mDCs and of TNF-α^+^ and IFN-α^+^ pDCs in elderly[16]. In contrast, they also showed higher percentages of IL-6^+^ mDCs and TNF-α^+^ pDCs in elderly. A possible explanation could be that Panda et al. used a mixed population of male and female subjects, with 70% females in the young population and 41% females in the old population. It could be speculated that older males have a higher basal cytokine production by mDCs compared to women, as is known for IL-6 levels in blood [36]. This increased basal cytokine production by both mDCs and pDCs of elderly, although only confirmed for IFN-α^+^ pDCs in this study, could contribute to the increased level of IL-6 and TNF-α in serum observed in general in inflammageing[8].

Although in general the elderly suffer from inflammageing, which is expressed by an increased basal cytokine production, elderly often have reduced cytokine responses after TLR stimulation as reviewed by Kollman et al and Shaw et al[7,37]. In this paper, we focused on the differences between pDC and mDC responsiveness in elderly and young women.

After TLR7/8 stimulation, elderly women had less IL-6^+^ and TNF-α^+^ pDCs and less IFN-α^+^ pDCs. However, upon other TLR stimulations elderly had more IFN-α^+^ pDCs, albeit with very low percentages. This difference in reactivity could partly be explained by reduced TLR4 expression. However, this does not account for TLR2 and TLR9, of which the expression was even increased in elderly, see S4 Fig. Our findings confirm other studies in which pDCs were stimulated with influenza or West Nile Virus (TLR7) [17,38], R848 (TLR7/8) [24,38,39] or CpG (TLR9) [17,39]. In these studies, no differences were observed in the amount of IFN-α produced per cell[17]. In contrast to our finding that TLR9 expression in pDCs was increased, Panda et al. observed a similar TLR9 expression on pDCs between old and young adults, and reduced TLR7 mRNA expression in elderly, whereas Garbe et al. found reduced TLR9 expression upon ageing[16,26].

Upon TLR stimulation, mDCs of elderly women had a similar percentage of IL-6^+^ mDCs and less TNF-α^+^ mDCs for all TLR stimulations compared to young women. The percentage of IFN-α+ mDCs was higher in elderly after TLR1/2 stimulation, but lower after TLR9 stimulation. In contrast to Krug et al., IFN-α^+^ mDCs were observed upon CpG stimulation [40]. For both TLR1/2 and TLR9 stimulation, the percentage of IFN-α^+^ mDCs was very low and therefore the biological relevance is debatable. In the study of Panda et al. mDCs of young people were more responsive to stimulation with TLR1/2, TLR2/6, TLR5 and TLR7/8 than mDCs from elderly, which is confirmed in our study[16]. This suggest that mDCs of elderly are less able to respond to viral and bacterial infections, leading potentially to more severe infections in elderly.

Within the elderly study group there were some individuals of whom the mDCs did not produce IL-6 or a little IL-6 upon stimulation with PAM, LPS, R848 and CpG, the so-called ‘non-responders’. There seemed to be a correlation between BMI and low IL-6 cytokine production by mDCs as the non-responding group had on an average a higher BMI (25.3 kg/m^2^) compared to the responding group(23.6 kg/m^2^). From literature some examples of a correlation between a high BMI and an increased cytokine production are known, but no decreased cytokine production [41,42]. Therefore more research is needed to investigate this potential correlation.

Of all TLR expression we measured on mDCs, only TLR2 expression was significantly higher in elderly compared to young women (S5 Fig). In contrast, Panda et al measured a higher TLR 1, 2, 3 and 8 expression on mDC by flow cytometric analysis in young adults, while Jing et al. observed comparable TLR2 and TLR4 expression between young adults and elderly [16,17].

So although elderly have comparable relative numbers of mDCs compared to young adults, their mDCs are less responsive to TLR stimulation, which cannot be explained by TLR expression. These phenomena may underlie part of the observed immunosenescence upon ageing.

In serum of elderly women, increased levels of sICAM, sVCAM, TNF-α and IL-10 were detected compared to young women. For TNF-α this is confirmed by many studies [8,32,43,44], although not all[45–48]. An increased level of IL-6 is often observed[8,44,45,47–50], while in our study IL-6 was slightly higher in elderly but not significantly enhanced, like in the study of Beharka et al [51]. Some studies observed no difference between old and young individuals for serum levels of IL-6 and IL-10 [32,52] and TNF-α [48,52]. For IL-10, several studies do not detect differences in serum levels upon ageing [46,47,50,52], while in our study IL-10 was enhanced in elderly women. A trend was observed of increased levels of IL-1Ra in serum, which is in line with other studies[45,53]. An increased level of IL-1Ra could be considered beneficial; IL-1Ra is a natural inhibitor of IL-1β as they bind the same IL-1β-receptors, but IL-1Ra binding does not lead to receptor activation [54]. High concentrations of TNF-α in serum have been associated with Alzheimer disease, atherosclerosis and frailty[44,55]. TNF-α together with IL-1β induces IL-6 production, while the three cytokines together induce the production of CRP, leading to inflammatory responses in multiple sites of the body[2,44]. A potential explanation for not observing a significant increase in IL-6 and CRP concentration in serum in elderly in this study, might be related to the low levels of IL-1β that were detected as well[46]. An explanation for the increased levels of IL-10 and TNF-α in serum of elderly women could be the decreased levels of estrogen. Estrogen used as hormone replacement therapy in elderly women has been shown to reduce IL-10, IL-6 and TNF-α levels in serum, as reviewed by Giefing-Kroll et al[56].

Next to cytokines, the adhesion molecules soluble ICAM (sICAM) and sVCAM-1 were elevated in elderly compared to young adults, which is in line with other studies [50,57,58]. Soluble forms of ICAM and VCAM are shed from the cell surface, and their expression is connected to migration of immune cells towards arthero-sclerotic plaques and inflammatory lesions[58]. It has been suggested that sICAM and sVCAM expression is activated by oxidative stress, which is supposed to increase by age[58]. Antioxidant supplementation, which is supposed to mimic the antioxidant effect of estrogen in postmenopausal women, resulted in reduced levels of sICAM and sVCAM [59]. Therefore the increase in both sICAM and sVCAM in our study in the postmenopausal group of women could partly be explained by the loss in estrogen production, which is observed in general. However, increased levels of sICAM and sVCAM are also observed in mixed ageing population[50,58]. Altogether, there is an increase in pro-inflammatory markers in serum of elderly women, confirming the concept of inflammageing. This inflammageing may be related to the reduction in estrogen levels after menopause.

Recent hypotheses have suggested that the decreased response to TLR stimuli in elderly people and the enhanced steady state production of cytokines by blood cells of elderly people may both linked to miRNAs[60]. Interestingly, miRNAs seem to play an important role in TLR signaling [60] and these miRNAs (e.g. miRNA-21; miRNA-126;miRNA-146) are modulated during ageing.[61]. MiRNA-21 increases upon ageing, while miRNA-146 decreases, although concentrations in serum of both miRNAs can be decreased by estrogen hormone replacement therapy (HRT) in postmenopausal women[62]. So, if ageing affects miRNA expression, leading to dysregulation of TLR function (increased pro-inflammatory cytokines), and if miRNA can also inhibit TLR function, the balance of inhibitory and stimulatory miRNA might play a role in both processes. This would be an interesting topic to study in-depth in future gerontological studies.

In this study differences between elderly and young women are observed. It should be stated that this was a cross sectional study. As we did not follow-up the group of elderly women over time, it is difficult to pinpoint a reason for differences found. Nevertheless, it remains a question whether differences in ageing immune systems compared to younger immune systems is a bad thing in itself. One would expect that a lifetime of exposure to environmental factors such as infections and vaccinations would result in an experienced immune system with less naive T cells [21], less CD34 progenitor cells[32,35] and thereby changed numbers in dendritic cells as well. In steady state, it might be a positive situation that pDCs and mDCs of elderly do not produce many cytokines, as it would increase the levels of pro-inflammatory cytokines in serum even further. Reduced responsiveness to pathogens, as mimicked by stimulation by TLR agonists, can be beneficial in case of severe infections of influenza, as immune-pathological effects are reduced [63]. As long as an individual eventually can clear the virus, this does not have to be a problem. It does become a problem when pathogens cannot be cleared. This is often the case in elderly when it comes to mortality caused by influenza infections[10].

## Conclusion

In this study, elderly women were shown to have relatively lower pDCs frequencies, comparable relative mDC frequencies, lower basal production of cytokines by pDCs and mDCs in steady state, and increased markers involved in inflammation in serum, compared to young adult women. Especially after TLR7/8 stimulation, pDCs and mDCs of elderly are less responsive compared to young adult women. However, no direct link was observed between intracellular immune responses by pDCs and mDCs and pro-inflammatory cytokines in serum. This study confirms and extends the knowledge about immunosenescence and inflammageing on innate immunity in elderly women.

## Supporting information

S1 Fig **Intracellular cytokine production of pDCs** by a donor from the elderly population (171/002) (A) or a donor from the young adult population (171/young/3) (B) upon TLR 7/8 stimulation with R848.
(PDF)

S2 Fig **Intracellular cytokine production by pDCs** of a control sample of a young adult woman (A) and an elderly woman (B); mDC/pDC backbone with isotype controls for IFN-α, IL-6 and TNF- α
(PDF)

S3 Fig **Percentage of pDCs (upper graph) and mDCs (lowergraph) in steady state (RPMI) and upon TLR stimulation in elderly (white) and young adults (black squares).** Data shown as 5–95% whisker plots, outliers with >2SD based on transformed data were removed. Elderly n = 30; young n = 15 donors. Statistics were done with logit transformed data using MANOVA with a pairwise comparison and a bonferroni correction. *p<0.05; **p<0.01; *** p<0.001.
(TIF)

S4 Fig TLR expression on pDCs of elderly and young women measured ex vivo. Statistical analysis was done using a MANOVA with a bonferroni correction. * p<0.05; ** p<0.01; ***p<0.001.
(TIF)

S5 Fig TLR expression on mDCs of elderly and young women measured ex vivo. Statistical analysis was done using a MANOVA with a bonferroni correction. * p<0.05; ** p<0.01; ***p<0.001.
(TIF)

S6 Fig Data master file of all experiments. For all measurements, the original and transformed data are showed and outliers (>2SD difference from the mean) based on transformed data are highlighted in orange.
(XLSX)
