# Supplementary figures and images for: Plasmacytoid dendritic cell and myeloid dendritic cell function in ageing: A comparison between elderly and young adult women
Source: PLoS One. 2019 Dec 12;14(12):e0225825. doi: 10.1371/journal.pone.0225825 (PMC6907850; doi:10.1371/journal.pone.0225825)

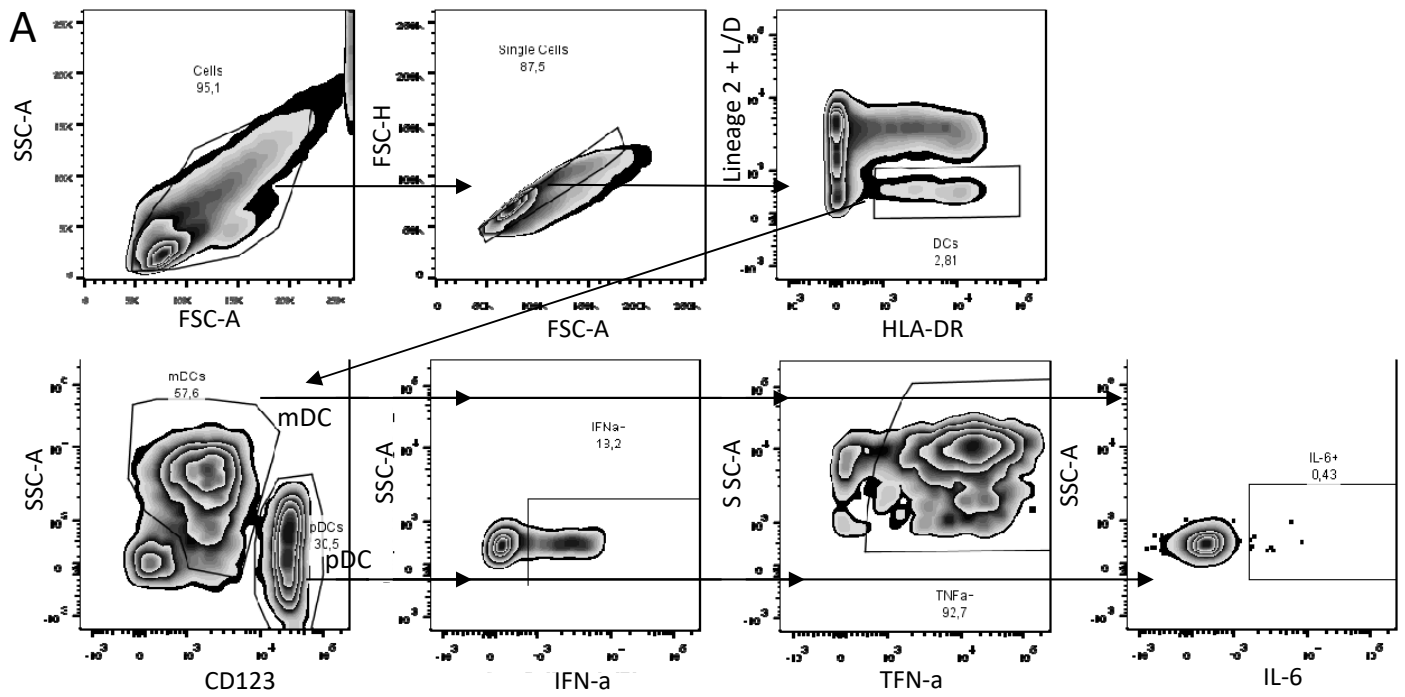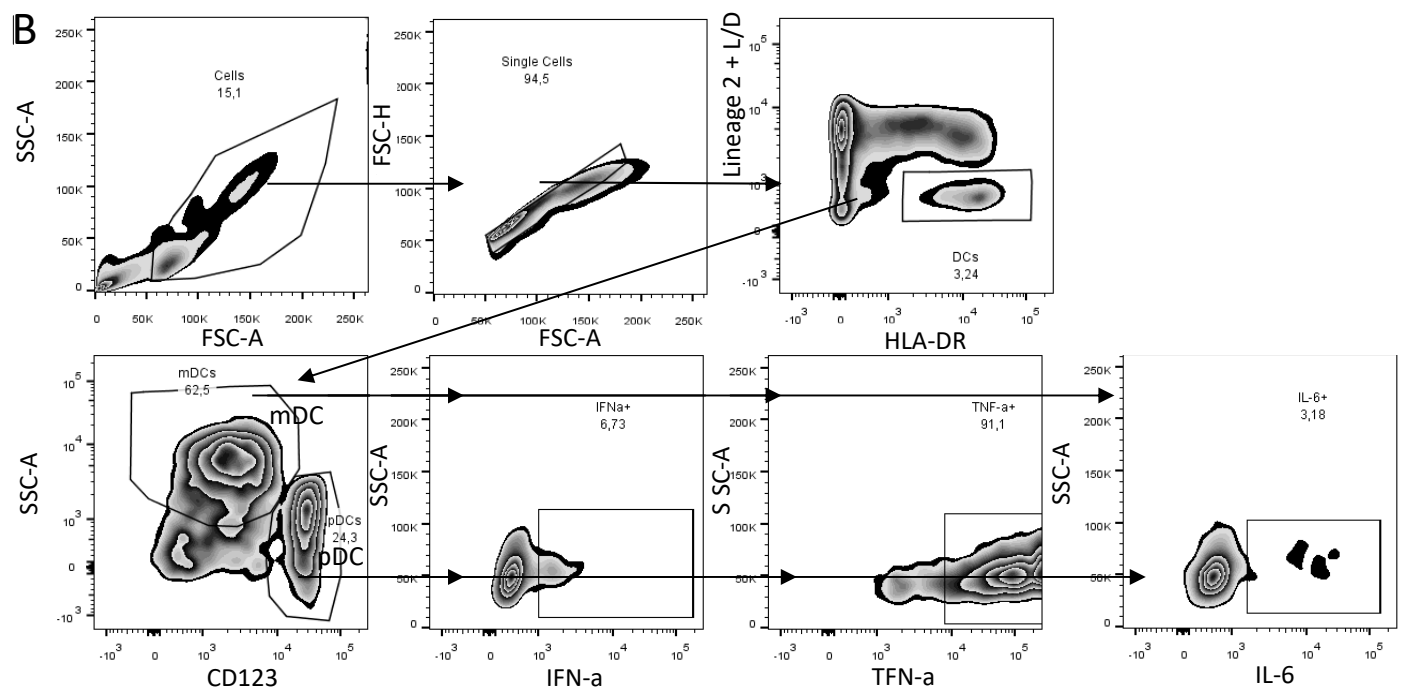

Supplement: S1 Fig — Intracellular cytokine production of pDCs by a donor from the elderly population (171/002) (A) or a donor from the young adult population (171/young/3) (B) upon TLR 7/8 stimulation with R848. (PDF) [file pone.0225825.s001.pdf]

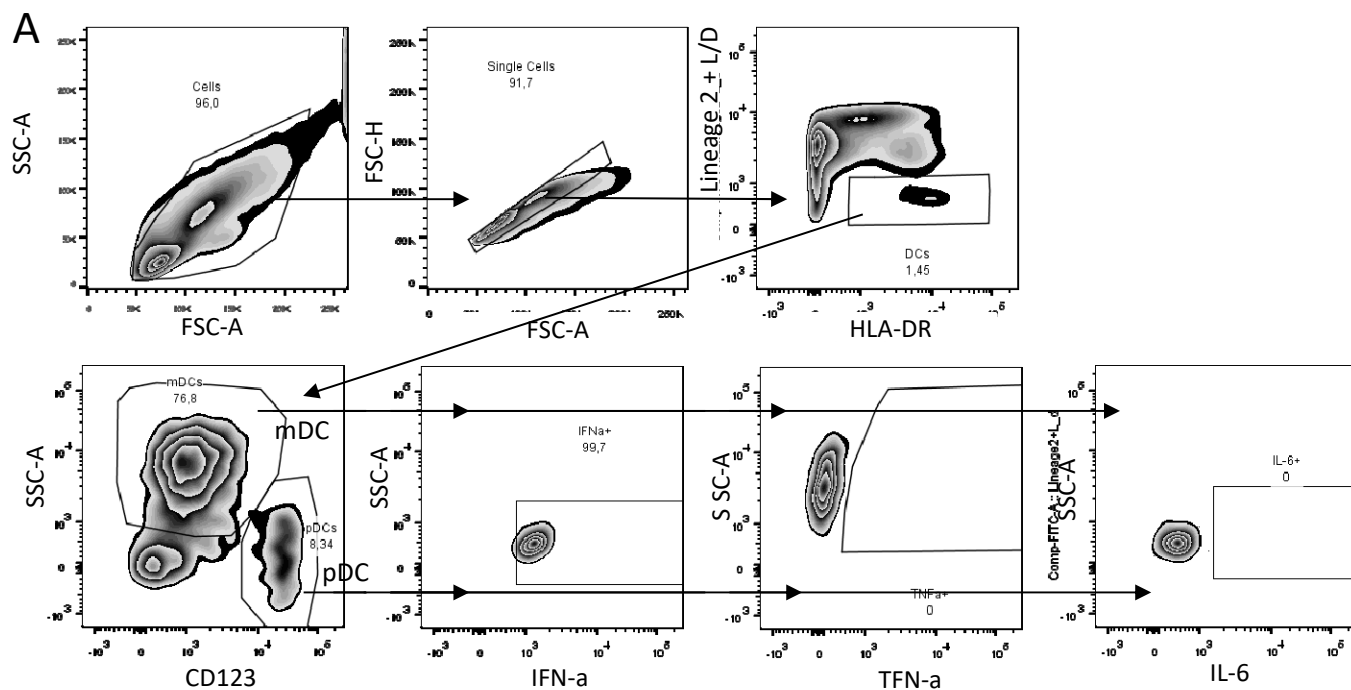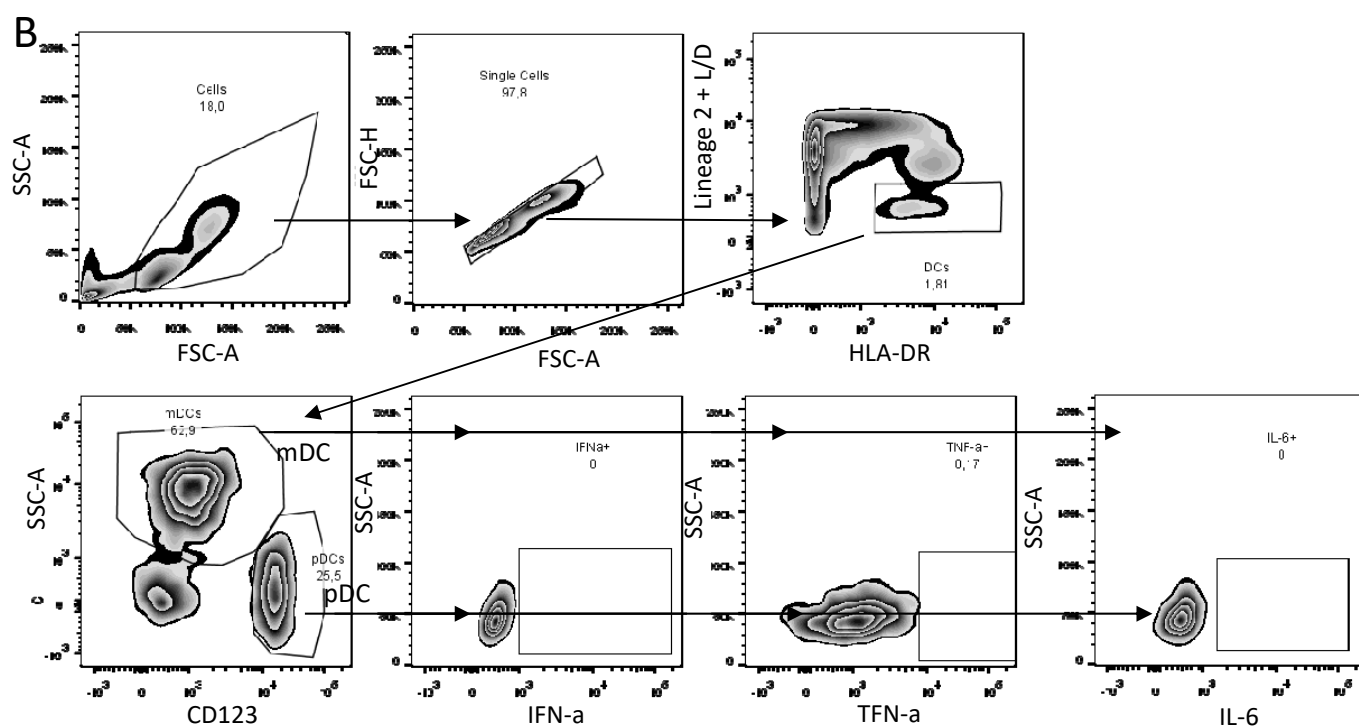

Supplement: S2 Fig — Intracellular cytokine production by pDCs of a control sample of a young adult woman (A) and an elderly woman (B); mDC/pDC backbone with isotype controls for IFN-α, IL-6 and TNF- α (PDF) [file pone.0225825.s002.pdf]

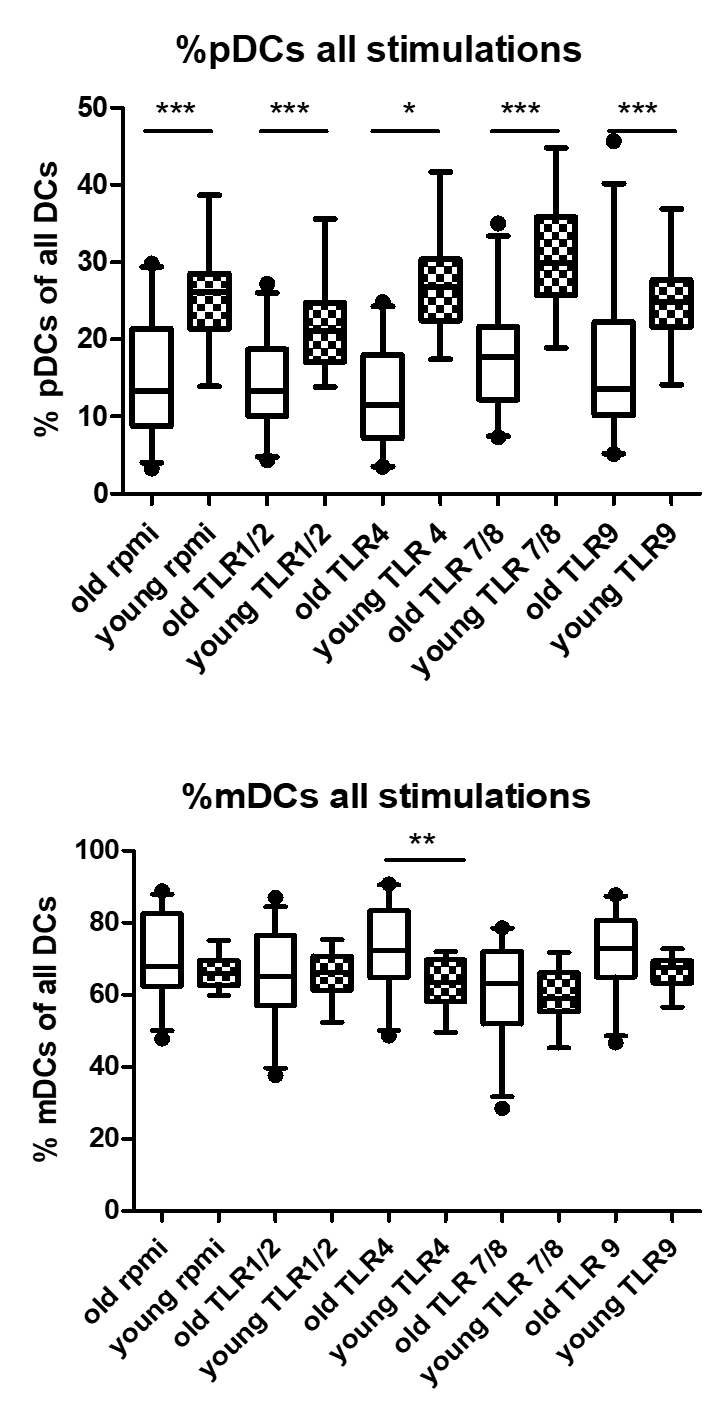

Supplement: S3 Fig — Percentage of pDCs (upper graph) and mDCs (lowergraph) in steady state (RPMI) and upon TLR stimulation in elderly (white) and young adults (black squares). Data shown as 5–95% whisker plots, outliers with >2SD based on transformed data were removed. Elderly n = 30; young n = 15 donors. Statistics were done with logit transformed data using MANOVA with a pairwise comparison and a bonferroni correction. *p<0.05; **p<0.01; *** p<0.001. (TIF) [file pone.0225825.s003.tif]

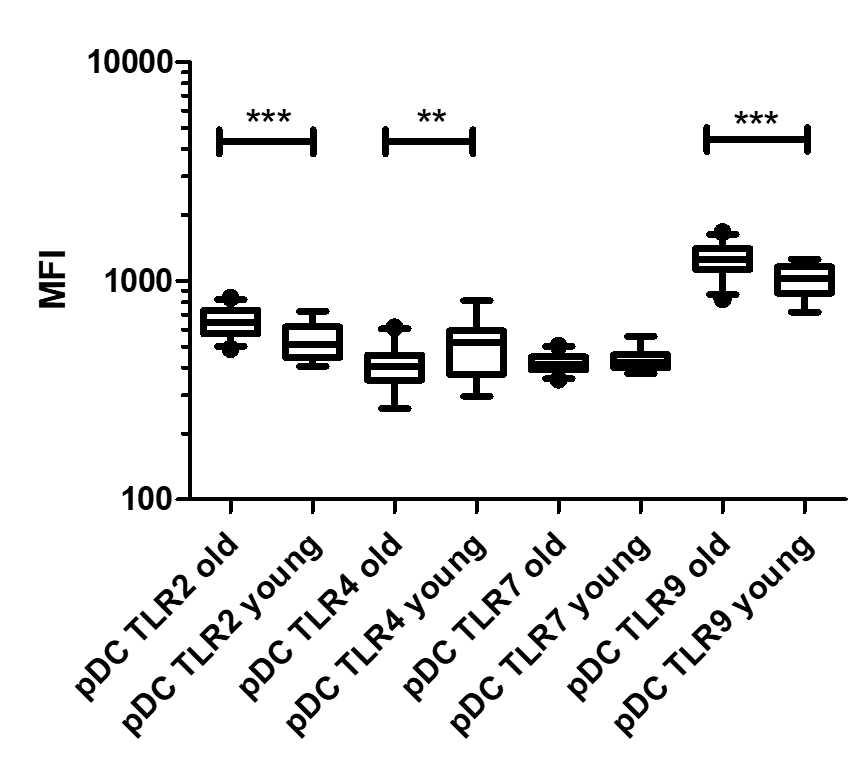

Supplement: S4 Fig — Statistical analysis was done using a MANOVA with a bonferroni correction. * p<0.05; ** p<0.01; ***p<0.001. (TIF) [file pone.0225825.s004.tif]

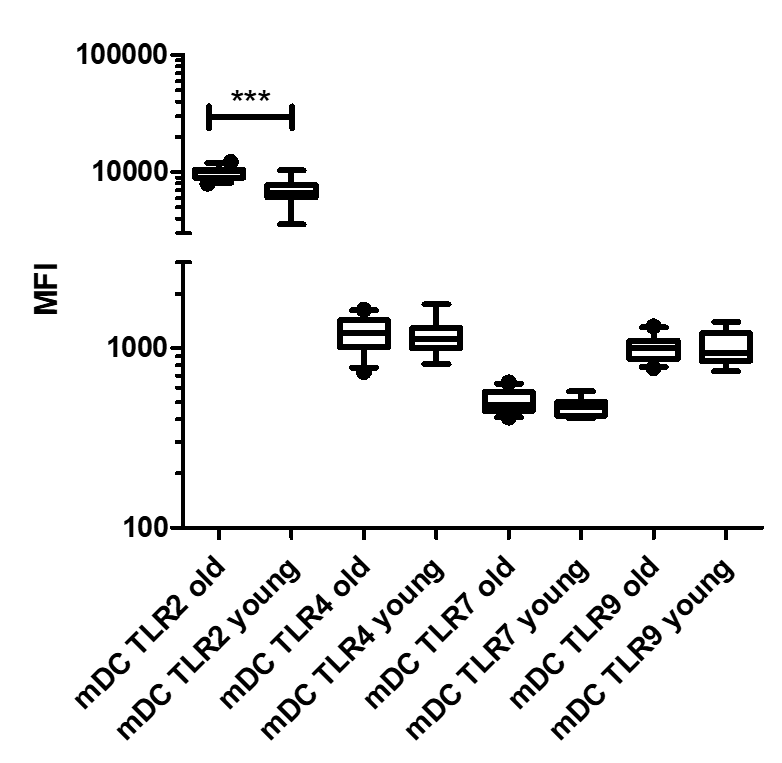

Supplement: S5 Fig — Statistical analysis was done using a MANOVA with a bonferroni correction. * p<0.05; ** p<0.01; ***p<0.001. (TIF) [file pone.0225825.s005.tif]
